# Supplementary material for: Economic model to examine the cost-effectiveness of FlowOx home therapy compared to standard care in patients with peripheral artery disease
Source: PLoS One. 2021 Jan 14;16(1):e0244851. doi: 10.1371/journal.pone.0244851 (PMC7808667; doi:10.1371/journal.pone.0244851)
Supplement: S1 Appendix — (DOCX) [file pone.0244851.s001.docx]

# SI1 Appendix: Model parameters

**Table A. Unit costs**

| **Description** | **Unit Cost (£)** | **Description** | **Reference*** |
| --- | --- | --- | --- |
| **Nominal Care** | | | |
| Vascular specialist nurse | 71 per visit | Mild = 1 per quarter  Prog = 1 per month  Sev = 2 per month | (33) |
| **Standard Care**  **Mean cost of medication/treatment** | | | |
| Aspirin | 11.07 per yr |  | (49) |
| Statins | 18.20 per yr |  | (49) |
| Non-Aspirin antiplatelet therapy | 41.37 per yr |  | (49) |
| Pregabalin/Gabapentin | 56.63 per Rx |  | (50) |
| Cost of wound care and treatment^a^ | 265 per wk |  | (11) |
| Cholesterol test | 3 per test |  | (49) |
| Angioplasty treatment | 3731.09 per yr |  | (33) |
| Nurse visit | 25 per 30min |  | (11) |
| **Flow-Ox** | | | |
| Rental Cost | 15 per day | Mild = 1 per day for 3 months  Prog = 1 per day for 3 months  Sev = 1 per day for 3 months | Direct communication with Otivio (FlowOx™manufacturer) |
| **Amputation** | | | |
| Average cost of minor amputation | 8961.52 |  | (33) |
| Average cost of major amputation | 16094.71 |  | (33) |
| Cost of rehabilitation | 438.49 |  | (33) |
| Cost of post amputation and prosthesis | 3061.16 |  | (11) |

*see reference list in main test for full reference

^a^ includes treatment costs and cost of analgesics and dressings

**Table B. Unit cost per health state**

| **Description** | **Dosage**^a^ | **Unit Cost (£)** | **Cost per State** |
| --- | --- | --- | --- |
| **Mild** | | | |
| Vascular Specialist Nurse | 1 visit per quarter | 71 | **143.09** |
| Nurse/dietician/Supervised exercise | 2 or 3 appointments (30min) | 25 |  |
| Cholesterol test |  | 3 |  |
| medication | Per yr | 25.73 |  |
|  | | | |
| **Progressive** | | | |
| Vascular Specialist Nurse | 2 visit per quarter | 71 | **828.43** |
| Nurse/dietician/Supervised exercised | 3 appointments (30min) | 25 |  |
| Cholesterol test |  | 3 |  |
| MRI |  | 138 |  |
| Medication |  | 33.90 |  |
| Treatment^b^ |  | 461.96 |  |
|  | | | |
| **Severe** | | | |
| Consultant Led Clinic Attendance |  | 253 | **1767.33** |
| Nurse | 1 per wk | 25 |  |
| Dressing and other consumables | 1 per wk | 3 |  |
| Angiogram |  | 374 |  |
| Angioplasty/treatment^b^ |  | 767.17 |  |
| Medication | Per yr | 58.41 |  |
|  | | | |
| **Minor Amputation** | | | |
| Minor Amputation & Rehabilitation | per yr | 8961.52 | **2254.98** |
| Medication | Per yr | 58.41 |  |
|  | | | |
| **Multiple Minor Amputation** | | | |
| Multiple Minor Amputation & Rehabilitation | per yr | 11233.52 | **2822.98** |
| Medication | Per yr | 58.41 |  |
|  | | | |
| **Major Amputation** | | | |
| Major Amputation | per yr | 16094.71 | **4913.19** |
| Rehabilitation | per yr | 438.49 |  |
| Medication | Per yr | 58.41 |  |
| Cost of Post Amputation/Prosthetics | per yr | 3061.16 |  |
|  | | | |
| **Multiple Major Amputation** | | | |
| Multiple Major Amputation | per yr | 18888.54 | **5611.65** |
| Rehabilitation | per yr | 438.49 |  |
| Medication | Per yr | 58.41 |  |
| Cost of Post Amputation/Prosthetics | per yr | 3061.16 |  |

^a^ Dosage and unit were agreed upon by expert opinion.

^b^ Various types of treatment were observed, hence mean values were applied

**Table C. Health state utilities**

| **Description** | **Mean Value** | **Reference*** |
| --- | --- | --- |
| Mild | 0.81 | (51) |
| Progressive | 0.66 | (51) |
| Severe | 0.44 | Primary data |
| Minor Amputation | 0.61 | Primary data |
| Major Amputation | 0.31 | Primary data |
| Recover | 0.70 | (52) |

* see reference list in main test for full reference

**Table D. Transition probabilities**

| **Description** | **Annual transition probabilities** | **Reference*** |
| --- | --- | --- |
| **Mild** | | |
| Prog | 0.021 | (51) |
| Sev | 0.004 | (51) |
| Dead | 0.02 | (51) |
| **Progressive** | | |
| Sev | 0.064 | (53) |
| Minor amputation | 0.032 | (54) |
| Major amputation | 0.022 | (54) |
| Dead | 0.095 | (3) |
| **Severe** | | |
| Minor amputation | 0.22 | (55-57) |
| Major amputation | 0.16 | (55-57) |
| Dead | 0.12 | (56, 57) |
| **Minor Amputation** | | |
| Rec | 0.08 | (29) |
| Major amputation | 0.15 | (30) |
| Dead | 0.10 | (58) |
| **Major Amputation** | | |
| Rec | 0.40 | (29) |
| Dead | 0.23 | (59) |
| **Other** | | |
| Relative risk of disease progression | 0.77 | NA |
| Relative benefit | 1.1 | NA |

*see reference list in main test for full reference

**Table E. Detailed cost per state**

| **Unit Description** | | **Unit cost (£)** | **Cost (£) per year^b^** | **Total cost (£) per year** |
| --- | --- | --- | --- | --- |
| **Mild** | | | | |
| Average cost of medication | Aspirin | 11.07 per yr | 25.73 | **572.36** |
|  | Statin | 18.20 per yr |  |  |
|  | Non-Aspirin antiplatelet therapy | 18.20 per yr |  |  |
| Wound care/ treatment |  | 265 per visit | 546.64 |  |
| Cost of cholesterol test^a^ |  | 3 per test |  |  |
| **Progressive** | | | | |
| Average cost of medication | Aspirin | 11.07 per yr | 33.90 | **3313.71** |
|  | Statin | 18.20 per yr |  |  |
|  | Non-Aspirin antiplatelet therapy | 18.20 per yr |  |  |
|  | Pregabalin | 56.63 per Rx |  |  |
| Wound care/ treatment |  | 265 per wk | 3279.82 |  |
| Cost of cholesterol test^a^ |  | 3 per test |  |  |
| **Severe** | | | | |
| Average cost of medication | Pregabalin/ Gabapentin | 56.63 per Rx | 58.41 | **7069.31** |
| Angioplasty treatment |  | 3731.09 | 3731.09 |  |
| Wound care/ treatment |  | 265 per wk | 3279.82 |  |
| **Minor Amputation^c^** | | | | |
| Cost of minor amputation |  | 8961.52 | 8961.52 | **9019.93** |
| Cost of medication | Pregabalin/ Gabapentin | 56.63 per Rx | 58.41 |  |
| **Major Amputation^c^** | | | | |
| Cost of major amputation |  | 16094.71 | 16094.71 | **19652.77** |
| Cost of rehabilitation |  | 438.49 | 438.49 |  |
| Cost of medication | Pregabalin/ Gabapentin | 56.63 per Rx | 58.41 |  |
| Post-amputation care and prosthesis |  | 3061.16 | 3061.16 |  |

1. Cost of cholesterol test replaces cost of ulcer dressing.
2. All cost were updated using the inflation index (36)
3. Annual cost given, but does not repeat across multiple years as this is a transitory state
